# Impact of D-Dimer for Prediction of Incident Occult Cancer in Patients with Unprovoked Venous Thromboembolism

**DOI:** 10.1371/journal.pone.0153514

**Published:** 2016-04-13

**Authors:** Donghee Han, Bríain ó Hartaigh, Ji Hyun Lee, In-Jeong Cho, Chi Young Shim, Hyuk-Jae Chang, Geu-Ru Hong, Jong-Won Ha, Namsik Chung

**Affiliations:** 1 Department of Internal Medicine, Division of Cardiology, Severance Hospital, Yonsei University College of Medicine, Seoul, Republic of Korea; 2 Dalio Institute of Cardiovascular Imaging, New York-Presbyterian Hospital and the Weill Cornell Medical Center, New York, New York, United States of America; Maastricht University Medical Center, NETHERLANDS

## Abstract

**Background:**

Unprovoked venous thromboembolism (VTE) is related to a higher incidence of occult cancer. D-dimer is clinically used for screening VTE, and has often been shown to be present in patients with malignancy. We explored the predictive value of D-dimer for detecting occult cancer in patients with unprovoked VTE.

**Methods:**

We retrospectively examined data from 824 patients diagnosed with deep vein thrombosis or pulmonary thromboembolism. Of these, 169 (20.5%) patients diagnosed with unprovoked VTE were selected to participate in this study. D-dimer was categorized into three groups as: <2,000, 2,000–4,000, and >4,000 ng/ml. Cox regression analysis was employed to estimate the odds of occult cancer and metastatic state of cancer according to D-dimer categories.

**Results:**

During a median 5.3 (interquartile range: 3.4–6.7) years of follow-up, 24 (14%) patients with unprovoked VTE were diagnosed with cancer. Of these patients, 16 (67%) were identified as having been diagnosed with metastatic cancer. Log transformed D-dimer levels were significantly higher in those with occult cancer as compared with patients without diagnosis of occult cancer (3.5±0.5 vs. 3.2±0.5, P-value = 0.009, respectively). D-dimer levels >4,000 ng/ml was independently associated with occult cancer (HR: 4.12, 95% CI: 1.54–11.04, P-value = 0.005) when compared with D-dimer levels <2,000 ng/ml, even after adjusting for age, gender, and type of VTE (e.g., deep vein thrombosis or pulmonary thromboembolism). D-dimer levels >4000 ng/ml were also associated with a higher likelihood of metastatic cancer (HR: 9.55, 95% CI: 2.46–37.17, P-value <0.001).

**Conclusion:**

Elevated D-dimer concentrations >4000 ng/ml are independently associated with the likelihood of occult cancer among patients with unprovoked VTE.

## Introduction

Venous thromboembolism (VTE) that includes deep vein thrombosis (DVT) and pulmonary embolism (PE) is closely linked with a higher burden of cancer. Foremost, cancer is known to provoke the onset of VTE, and reciprocally, VTE along with its complications are frequently involved in the poor prognosis observed among patients with cancer. Previous studies reported that up to 30% of all first VTE occurred in patients with cancer [1,2]. Provoked VTE is typically defined as VTE occurring in the presence of a risk factor such as a recognizable cancer, pregnancy, or major surgery. Conversely, unprovoked VTE is considered to occur in the absence of any discernable risk factors [3]. To this end, unprovoked VTE is considered the first manifestation in patients with occult cancer. Further still, prior studies have documented that the risk of cancer appeared to increase in patients presenting with unprovoked VTE, particularly during an early follow-up period [4–6].

D-dimer represents a biomarker that reflects the activation of hemostasis and fibrinolysis. In recent past, D-dimer has displayed high sensitivity for diagnosis of VTE and its related adverse outcomes [7–9]. Current guidelines recommend the use of measuring D-dimer in patients for the purpose of screening for suspected VTE [3]. Further still, elevated D-dimer levels have been shown to be present in those with malignancies, with the relationship between high D-dimer concentrations and the presence of cancer reported elsewhere [10–12]. While it appears elevations in D-dimer levels are independently linked with VTE as well as cancer, to date, the predictive value of D-dimer and its relationship with occult cancer in patients with VTE remains to be firmly established. We therefore aimed to evaluate whether D-dimer might serve as a useful tool for predicting occult cancer in patients who present with unprovoked VTE.

## Materials and Methods

### Study design and population

**Fig 1:**
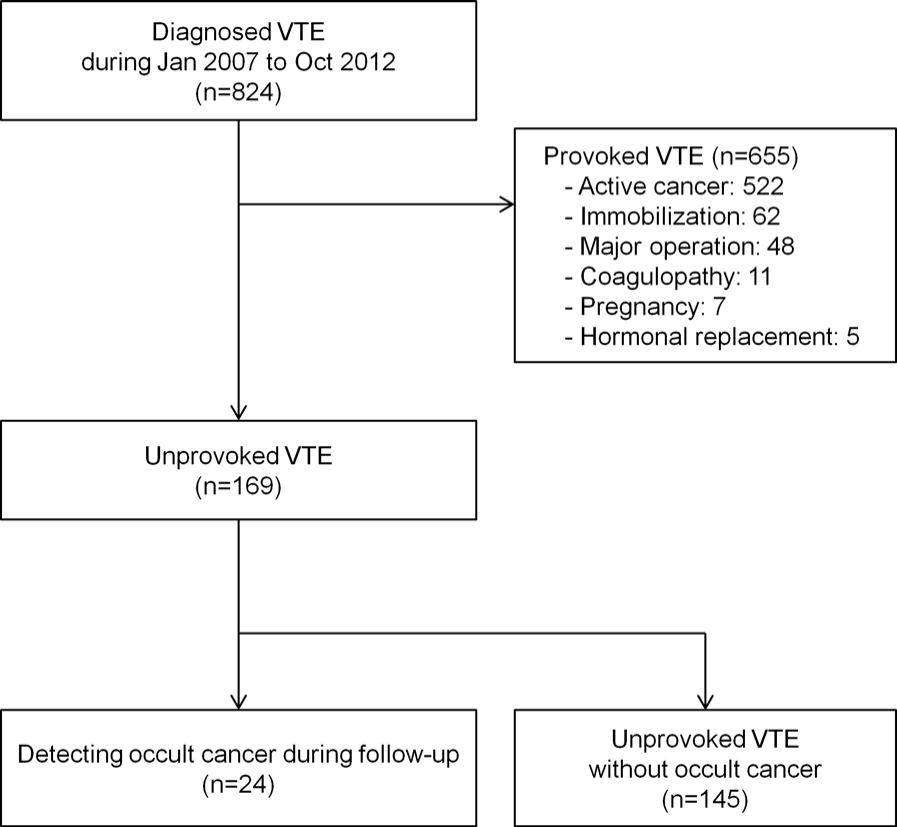
Flow chart of the study cohorts.

We retrospectively reviewed medical records of patients who were diagnosed with DVT or PE from January 2007 to October 2012, in a single medical center. A study flow chart of this study is displayed in in Fig 1. The study population was classified according to cause of VTE. Provoked VTE was defined due to the following factors based on current guidelines: known overt cancer, pregnancy including postpartum status, coagulopathy including thrombophilia, immobilization, major surgery, and the use of oral or hormone replacement therapy. Unprovoked VTE was defined as patients with VTE but in the absence of any cause. For the purpose of this study, 169 patients comprised the analytic sample in this study. This study was approved by the Institutional Review Board of Yonsei University, Severance Hospital, Seoul, Korea. Written informed consent was exempt by the board as this study was retrospective in design. Patient records/information was anonymized and de-identified prior to analysis.

### Data examination

We retrospectively reviewed patients’ medical records for information regarding age, sex, type of VTE, and comorbidities. The prevalence of hypertension was defined as a history of being diagnosed with hypertension, or use of antihypertensive medication. Prevalent diabetes mellitus was defined as having a history of being diagnosed with diabetes or the use of anti-glycemic medication. Coronary artery disease was defined as a history of being diagnosed for coronary artery disease. D-dimer was measured within 24 h of presenting to the medical department. The level of D-dimer was quantitatively assessed by HemosIL D-dimer HS for ACL TOP (Instrumentation Laboratory, Bedford, MA, USA), which is a latex-enhanced turbidimetric immunoassay with an imprecision ranging between 2.3% and 6.6% and a detection limit of 21 ng/ml. D-dimer was classified on the following D-dimer values: <2,000, 2,000–4,000, and >4,000 ng/ml, and also logarithmically transformed (D-dimer + 1) as a continuous measure.

### Study outcome

Patients were enrolled between January 2007 and October 2012, and followed through February 2015, until the last hospital visit from the first diagnosis date of VTE. During a median of 5.3 (interquartile range (IQR): 3.4–6.7) years study follow-up, patients were observed for an endpoint of detecting occult cancer. The primary origin and metastatic state of occult cancer in patients presenting with occult cancer was assessed. Data regarding the incidence of cancer events during the study follow-up period were obtained by review of the medical records.

### Statistical methods

Continuous variables are expressed as mean ± standard deviation (SD), and categorical variables are reported as counts with proportions. For the purpose of the study endpoint, our sample was dichotomized according to those with detected occult cancer (i.e., indicator = 1) or those without (i.e., indicator = 0). Comparisons between patient groups were performed by use of Student’s t-test for continuous variables and Pearson’s chi square test for categorical parameters. The incidence rate of occult cancer was calculated and compared according to D-dimer categories, pre-specified age groups, gender, and type of VTE. Multivariable Cox regression analysis reporting hazard ratios (HR) with 95% confidence intervals (CI) was employed to examine the risk of occult cancer according to D-dimer categories, adjusting for age, gender, and type of VTE. The diagnostic performance of D-dimer levels >4,000 for predicting occult cancer were assessed by calculating sensitivity, specificity, negative predictive value (NPV), and positive predictive value (PPV) metrics. A p-value <0.05 was considered statistically significant. All statistical analyses were performed using STATA Version 13 (StataCorp LP, College Station, TX, USA).

## Results

### Frequency and distribution of occult cancer in VTE patients

In Table 1, of the 169 VTE patients, 24 (14.2%) were subsequently diagnosed with occult cancer. The incidence of occult cancer per 100 person years was 3.1 (95% CI: 2.1–4.7). The median duration for detection of cancer after diagnosis of VTE was 6.5 (IQR 2–14) days. Overall, 21 (88%) of these patients were diagnosed with cancer during initial hospitalization, with the remaining 3 (12%) patients diagnosed with cancer following discharge after first admission. The median duration to diagnosis for 3 patients who were diagnosed with cancer after being discharged from hospital was 104 days. Of those detected with occult cancer, 16 (66.7%) patients presented with a metastatic state at cancer diagnosis. Further details of these patients’ characteristics are outlined in S1 Table.

**Table 1:** Characteristics of patients with VTE-related occult cancer.

	Number (%)
**No. diagnosed during 1**^**st**^ **admission**	21 (87.5)
**Diagnosis duration, days, median [IQR]**	6.5 [2–14]
**Cancer origin**	
**Stomach**	5 (20.8)
**Colon**	4 (16.7)
**Lung**	4 (16.7)
**Ovary**	2 (8.3)
**Biliary**	2 (8.3)
**Lymphoma**	2 (8.3)
**Thyroid**	2 (8.3)
**etc**^**a**^	3 (12.5)
**Metastatic state at cancer diagnosis**	16 (66.7)

VTE, venous thromboembolism; IQR, interquartile range; etc, et cetera

^a^1 patient each in sarcoma, thymoma, and unknown origin

### D-dimer for predicting occult cancer

Clinical characteristics of patients with unprovoked VTE according to the presence and absence of occult cancer are shown in Table 2. No significant differences in age, sex, comorbidities, or type of VTE were observed between groups. D-dimer and Log transformed D-dimer were significantly higher in patients with occult cancer as compared with those without.

**Table 2:** Baseline characteristics between patients with and without occult cancer.

	Without occult cancer (n = 145)	With occult cancer (n = 24)	P-value
**Age, years (mean ± SD)**	60.9 ± 15.7	55.7 ± 11.7	0.121
**Male, n (%)**	76 (52.4)	9 (37.5)	0.176
**Hypertension, n (%)**	63 (43.5)	6 (25.0)	0.089
**Diabetes mellitus, n (%)**	13 (9.0)	3 (12.5)	0.584
**Known CAD, n (%)**	9 (6.21)	0 (0)	0.210
**Type of VTE**			0.116
**Pulmonary embolism, n (%)**	54 (37.2)	13 (54.2)	
**Deep vein thrombosis, n (%)**	91 (62.8)	11 (45.8)	
**D-dimer, ng/ml (mean ± SD)**	2,834.7 ± 5,287.2	5,313.9 ± 5,533.2	0.036
**Log D-dimer (mean ± SD)**	3.2 ± 0.5	3.5 ± 0.5	0.009

SD, standard deviation; CAD, coronary artery disease; VTE, venous thromboembolism

**Fig 2:**
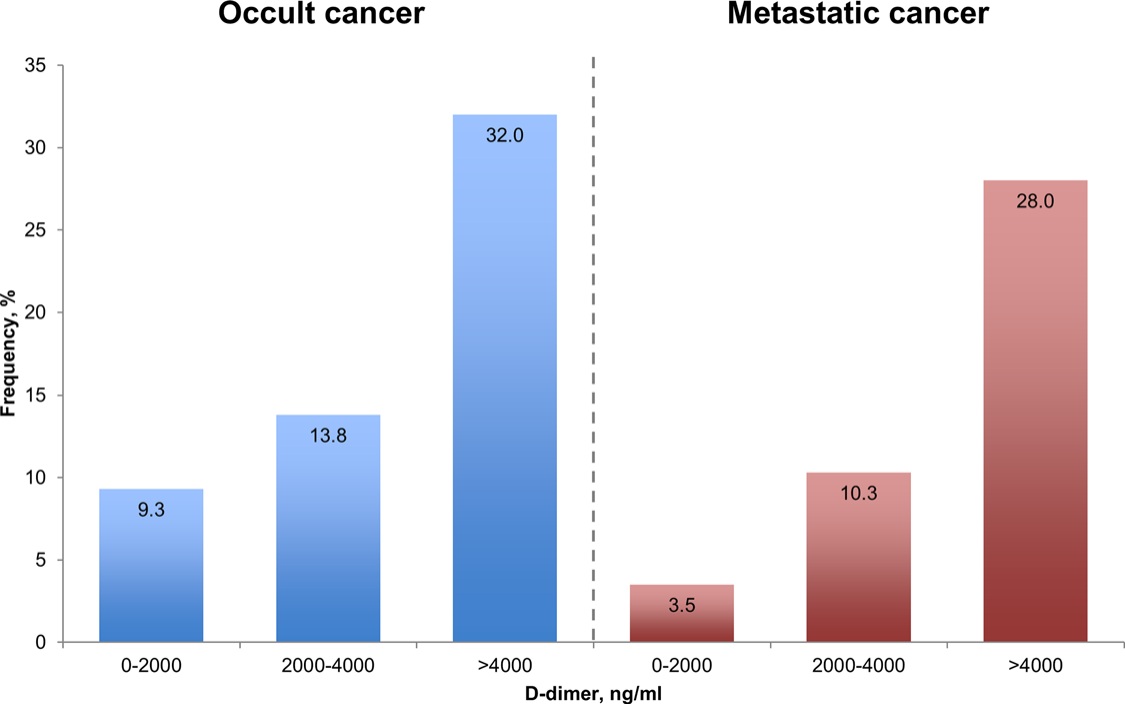
Incidence proportion of occult cancer and cancer with metastatic state according to quantitative level of D-dimer.

Fig 2 displays the incidence proportions of occult cancer, and metastatic state according to D-dimer categories. The proportion of occult cancer appeared higher in patients with baseline D-dimer levels >4,000 (e.g., 32.0%) when compared with the lower D-dimer levels of <2,000 (e.g., 9.3%) and 2,000–4,000 (e.g., 13.8%). Likewise, metastatic state at the time of cancer diagnosis was more predominant in patients with D-dimer levels >4,000.

In Table 3, depending on patient subsets, the incidence of occult cancer appeared higher in those <60 years of age, among women, for those with a pulmonary embolism, or when concentrations of D-dimer were >4000, as compared with their respective counterparts. Notably, the diagnostic performance of D-dimer levers >4,000 when screening for occult cancer yielded a sensitivity, specificity, PPV, and NPV of 33.3%, 88.3%, 32%, 88.9%, respectively.

**Table 3:** Incidence of occult cancer according to patient risk factors.

	Number of patients	Occult cancer cases	Incidence/100 person years
**Age group**			
**<60**	74	17 (23.0)	5.7
**60–70**	35	4 (11.4)	2.2
**>70**	60	3 (5.0)	1.0
**Gender**			
**Male**	85	9 (10.6)	2.1
**Female**	84	15 (17.9)	4.4
**VTE type**			
**DVT**	102	11 (10.8)	2.4
**PE**	67	13 (19.4)	4.3
**D-dimer group**			
**<2000**	86	8 (9.3)	1.8
**2000–4000**	58	8 (13.8)	3.3
**>4000**	25	8 (32.0)	9.1

VTE, venous thromboembolism; DVT, deep vein thrombosis; PE, pulmonary embolism

In Cox regression models, log transformed D-dimer was significantly associated with occult cancer, and a metastatic state of cancer (Table 4). Further, after adjusting for age, sex, and type of DVT, the risk of occult as well as metastatic state of cancer for patients with a D-dimer concentration >4,000 increased by more than 4-fold (HR: 4.12, 95% CI: 1.54–11.04, P-value = 0.005) and 9-fold (HR: 9.55, 95% CI: 2.46–37.17, P-value = 0.001), respectively, when compared with those with a D-dimer level <2,000.

**Table 4:** Cox regression for the likelihood of occult or metastatic cancer according to D-dimer.

	Occult cancer			Metastatic state of cancer		
	HR	95% CI	P value	HR	95% CI	P value
**Unadjusted**						
**Log D-dimer**	3.56	1.48–8.64	0.005	7.83	2.64–23.25	<0.001
**D-dimer groups**						
**<2000**	1	(Reference)		1	(Reference)	
**2000–4000**	1.58	0.59–4.22	0.357	3.17	0.79–12.66	0.103
**>4000**	4.02	1.51–10.71	0.005	9.33	2.41–36.15	0.001
**Adjusted**^a^						
**Log D-dimer**	3.98	1.58–10.02	0.003	10.06	3.08–32.87	<0.001
**D-dimer groups**						
**<2000**	1	(Reference)		1	(Reference)	
**2000–4000**	1.67	0.62–4.50	0.307	3.52	0.87–14.21	0.077
**>4000**	4.12	1.54–11.04	0.005	9.55	2.46–37.17	0.001

HR, hazard ratio; CI, confidential interval

^a^Adjusted for age, gender, and type of venous thromboembolism

## Discussion

In this retrospective observational study, we evaluated the predictive value of D-dimer for occult cancer in patients with unprovoked VTE. Our results indicated that increasing D-dimer levels were independently associated with a higher burden of occult cancer. Notably, a high D-dimer level was found to be a robust predictor of occult cancer in patients with unprovoked VTE.

### Risk of cancer in patients with VTE

The relationship between VTE and occult cancer has long been recognized. Although cancer most commonly provokes VTE, reciprocally, VTE may also lead to the diagnosis of occult cancer. Several epidemiological studies show that the risk of cancer is substantially elevated after diagnosis of VTE. One study evaluated the risk of cancer after diagnosis of DVT and PE using a population-based sample of 26,653 subjects, and demonstrated the risk of cancer was heightened during the first 6 months of follow-up [4]. Particularly, unprovoked VTE demonstrates a higher incidence of occult cancer compared with provoked VTE. The annual risk for new cancers was more than 2-fold higher in patients presenting with unprovoked compared with those with provoked VTE [5]. In a meta-analysis of 36 studies, the prevalence of diagnosed cancer with unprovoked VTE was 10.0% from baseline to 12 months [6], which is fitting with the current study results, whereby the proportion of diagnosed cancer in patients with unprovoked VTE was 14.2%.

### D-dimer and occult cancer

To date, studies evaluating the associations between D-dimer and occult cancer in patients with unprovoked VTE are sparse. Of few existing, Rege et al. reported that a low D-dimer level (i.e., <1,000 ng/ml) is a strong negative predictor for malignancy [13]. Although the latter study showed a significant association between D-dimer and malignancy in patients with VTE, those results did not comprehensively evaluate the association of D-dimer and occult cancer detected after diagnosis of VTE. In addition, Schutgens et al. also assessed the value of D-dimer in predicting cancer in patients with DVT [14]. In that study, higher D-dimer levels during the first few days of hospitalization were a strong indicator of an increased probability of occult forms of cancer. Our findings support as well as extend prior studies that investigated the relationship between D-dimer and occult cancer in patients with VTE. Our study was focused primarily on unprovoked VTE that had demonstrated a higher incidence of occult cancer compared with provoked VTE caused by other risk factors. Our study findings underline the possible association between high levels of baseline D-dimer and risk of occult cancer in patients with unprovoked VTE.

### Screening strategy for occult cancer in unprovoked VTE

Screening strategies for occult cancer in unprovoked VTE is an urgent issue that should be addressed in the clinical setting. In a previous study regarding screening for cancer in patients with unprovoked VTE, the findings were conflicting. Van Doormaal et al. proposed basic screening such as careful history and physical examination is the most optimal strategy screening method for occult cancer in these patients [15]. More recently, multicenter, randomized, controlled trial encompassing 854 patients showed that extended cancer screening within the abdomen and pelvis from CT did not provide a clinically significant benefit because of a low prevalence of occult cancer [16]. Current guidelines also suggest that the search for occult cancer following an episode of VTE might be confined to patients’ history, physical examination, basic laboratory tests, and chest X-ray [3]. By contrast, Piccioli et al. reported that extensive screening for occult cancer in patients with unprovoked VTE is warranted because early detection is likely to be associated with improved prognosis [17]. Taken together with the current diagnostic findings, high D-dimer should perhaps be considered during screening for occult cancer in these patient groups. Indeed, extensive screening for occult cancer may benefit those who present with higher D-dimer levels. Though, clearly, further studies are needed to assess the effectiveness for extensive screening of occult cancer in patients with unprovoked VTE on the background of high D-dimer levels.

### Limitations

The present study has some limitations that should be noted. The present study was retrospective and observational in nature. Therefore, the possibility of bias due to unmeasured confounding factors cannot be excluded. In addition, although we assessed some predictors of cancer risk (e.g., age, gender, and VTE), the retrospective nature of this study made it challenging to obtain information regarding other important potential risk factors for cancer (i.e., smoking), which should be considered in forthcoming investigations. This study was a single-center design and subjects comprised of Korean-Asian ethnicity only, which may have inferred a selection bias. Therefore, these findings may not be extrapolated to other ethnic origins, or specific Asian populations. Incidence of occult cancer was determined by review of medical records only, which might infer a potential possibility of missing patients with diagnosed cancer. Although we reviewed 824 patients diagnosed with VTE, only 169 patients were included as the analytic sample with 24 patients detected for having occult cancer. Particularly, the results of this study showed an inverse relationship between increasing age and incidence of occult cancer. This somewhat unusual association may be a consequence of a selection bias due to the relatively small sample size–though there may also have been the possibility of survivorship bias, possibly leading to an underestimation of a risk factor’s effect on disease risk [18]. Hence, our findings should be interpreted with caution, in light of the relatively low numbers of events, which likely explain the wide CIs observed. Information regarding other diagnostic tools for detecting occult malignancy was unavailable. Consequently, a reliable screening of occult malignancy in patients with unprovoked VTE was beyond the scope of this study, and could not be readily assessed.

## Conclusions

In conclusion, the current study demonstrated that higher D-dimer levels represent a strong independent indicator of increased risk of occult forms of malignancy in patients with unprovoked VTE. D-dimer levels may prove useful for augmenting cancer-screening strategies in patients with unprovoked VTE. Though additional studies are needed to support this contention.

## Supporting Information

S1 Table Clinical characteristics of patients diagnosed with cancer during follow-up. (DOCX)
