# Supplementary material for: Impact of D-Dimer for Prediction of Incident Occult Cancer in Patients with Unprovoked Venous Thromboembolism
Source: PLoS One. 2016 Apr 13;11(4):e0153514. doi: 10.1371/journal.pone.0153514 (PMC4830616; doi:10.1371/journal.pone.0153514)
Supplement: S1 Table — (DOCX) [file pone.0153514.s001.docx]

| S1 Table. Clinical characteristics of patients diagnosed with cancer during follow-up | | | | | | |
| --- | --- | --- | --- | --- | --- | --- |
| Age | Gender | Cancer diagnosis time since VTE diagnosis (day) | VTE type | Cancer organ | Clinical extension | Death (duration month) |
| 52 | F | 1 | PE | Ovary | Metastatic | Yes (18) |
| 56 | M | 4 | DVT | Stomach | Metastatic | Yes (5) |
| 49 | F | 1 | PE | Colon | Metastatic | Yes (2) |
| 74 | M | 7 | DVT | Stomach | Metastatic | Yes (13) |
| 59 | F | 12 | DVT | Biliary | Localized | Yes (3) |
| 51 | F | 104 | PE | Sarcoma | Metastatic | Yes (8) |
| 36 | F | 1 | PE | Stomach | Metastatic | Yes (14) |
| 62 | F | 16 | PE | Ovary | Metastatic | Yes (19) |
| 51 | F | 3 | PE | Lymphoma | Localized | Yes (3) |
| 69 | F | 156 | PE | Colon | Metastatic | Yes (7) |
| 47 | M | 3 | DVT | Thymoma | Metastatic | Yes (1) |
| 65 | F | 27 | DVT | Thyroid | Metastatic | Yes (1) |
| 76 | F | 11 | PE | Lung | Localized | No |
| 60 | M | 19 | PE | Lung | Metastatic | No |
| 48 | F | 6 | DVT | Lung | Metastatic | No |
| 52 | F | 11 | PE | Thyroid | Localized | No |
| 58 | M | 13 | DVT | Primary unknown | Metastatic | No |
| 46 | M | 1 | PE | Stomach | Metastatic | No |
| 39 | M | 1 | PE | Lung | Metastatic | No |
| 44 | F | 3 | DVT | Stomach | Metastatic | No |
| 59 | M | 9 | DVT | Colon | Localized | No |
| 42 | F | 14 | DVT | Lymphoma | Localized | No |
| 75 | M | 2 | DVT | Colon | Localized | No |
| 69 | F | 1 | PE | Biliary | Localized | No |
| VTE, venous thromboembolism; DVT, deep vein thrombosis; PE, pulmonary thromboembolism | | | | | | |
